# Sick-leave and help seeking among rescue workers after the terror attacks in Norway, 2011

**DOI:** 10.1186/s12245-015-0081-4

**Published:** 2015-08-19

**Authors:** Astrid Gjerland, May Janne Botha Pedersen, Øivind Ekeberg, Laila Skogstad

**Affiliations:** Department of Anesthesia, Intensive, Operation and Emergency, Baerum Hospital, Vestre Viken Hospital Trust, Drammen, Norway; Department of Surgery, Anesthesia and Obstetric, Ringerike Hospital, Vestre Viken Hospital Trust, Drammen, Norway; Department of Behavioural Sciences in Medicine, Institute of Basic Medical Sciences, Faculty of Medicine, University of Oslo, Oslo, Norway; Department of Acute Medicine, Research and Development, Oslo University Hospital, Postbox 4956 Nydalen, 0424 Oslo, Norway

**Keywords:** Disaster, Help seeking, Rescue workers, Sick-leave, Terror

## Abstract

**Background:**

Several studies have addressed psychological problems after terror attacks, especially among victims. Fewer have addressed possible health consequences among rescue workers involved with terror attacks. This study’s aim was to investigate the levels of sick-leave and psychological help seeking among rescue workers involved in the terror attacks in Norway on July 22, 2011, and to identify associations between sick-leave and background-, exposure- and work-related variables.

**Methods:**

This cross-sectional study included five groups of professional rescue personnel and one group of unaffiliated volunteers. The questionnaire was distributed approximately 10 months after the terror attacks, with a response rate of 61.3 % (*N* = 1790).

**Results:**

A total of 9.7 % of participants self-reported sick-leave. The rate varied from 2.4 % among police officers to 14.5 % among unaffiliated volunteers, *p* < .001. There were 0.0–1.2 % of the professionals who were on sick-leave for more than 2 weeks and 5.5 % among the unaffiliated volunteers. More unaffiliated volunteers (42.6 %) and psychosocial personnel (16.3 %) consulted a psychologist or psychiatrist compared to other groups (3–9 %), *p* < .001. General healthcare providers (OR 6.1), psychosocial personnel (OR 6.3) and unaffiliated volunteers (OR 5.7) were associated with sick-leave, together with unwanted stress reactions (OR 1.6) and starting work on July 22 (OR 1.6).

**Conclusions:**

A small minority of professional rescue workers reported sick-leave for more than 2 weeks, and few had sought psychological help. Unaffiliated volunteers reported more stress symptoms, longer sick-leave duration and more psychological help seeking. This group may benefit from more support.

## Background

Sick-leave and help seeking reflect physical, psychological and social functioning. Rescue workers are exposed to stressful events at times of disasters. This may put them at risk for health problems and absence from work. Few studies have addressed possible health consequences like sick-leave and help seeking among rescue workers in the aftermath of a terror attack. There are a variety of symptoms among rescue workers after traumatic events. After the explosion of a firework depot in the Netherlands in May 2000, long-term increases in psychological, musculoskeletal and respiratory problems were found [1]. This is in accordance with the most common adverse health effects after the World Trade Center (WTC) disaster in 2001 [2, 3]. Another study after the firework explosion found that rates of leave for psychological problems and other illnesses had returned to pre-disaster levels, whereas sick-leave for musculoskeletal and respiratory reasons remained elevated until 3 years post-disaster [4].

A systematic review and meta-analysis showed a 10 % worldwide prevalence of posttraumatic stress disorder (PTSD) among rescue workers, a rate higher than in the general population [5]. A higher prevalence of PTSD in ambulance personnel compared to fire-fighters and police officers were found. Probable PTSD has also been reported in rescue personnel after Western terror attacks: 4 % of ambulance staff after the 2005 London bombings [6], 13 % of fire-fighters after the Oklahoma City bombing in 1995 [7] and 5.4–14.4 % of fire-fighters and police officers after the WTC attacks [8]. A review showed that mental health needs for workers and volunteers after the terror attacks in USA 9/11 2001 ranged from little to no care to pharmacotherapy [9]. Other studies indicate that 80–90 % cope well after disasters [10, 11]. Even though it is important to measure the degree of symptoms, sick-leave may be a better measure of functioning, and such studies are lacking.

During the terror attacks in Norway on July 22, 2011, eight individuals were killed in the bombing of the Oslo government district and 69 were killed at the shooting at the youth camp on Utøya Island [12, 13]. More than 500 young people participated in the camp at Utøya and were psychologically affected by the shooting [14]. In addition to professional rescue workers, civil people who just happened to be at the terror site close to Utøya Island became rescuers. Terrorist attacks of this magnitude are still seldom in Western countries. This calls for studies of the level of health problems and possible differences between personnel with different tasks and roles.

This study’s aim was to investigate the levels of sick-leave and psychological help seeking among rescue workers involved in the terror attacks in Norway 2011, and to identify associations between sick-leave and background-, exposure- and work-related variables.

## Methods

This cross-sectional study targeted personnel involved in the rescue and healthcare services after the terror attacks in Norway 2011. The present paper is part of a larger study examining the challenges that the rescuers met [15]. There were two inclusion criteria: working (1) with victims and their relatives and (2) 1 day or more from July 22 until August 5, 2011.

All together, six groups participated: five professional groups and unaffiliated volunteers. The professional groups were as follows: healthcare providers, psychosocial personnel, police officers, fire-fighters and organized volunteers. The unaffiliated volunteers consisted of (1) civil people residing at the campsite or living on the landside facing Utøya Island. Some went by boat to rescue young people who were swimming from the island, even before the police had secured the area or took care of survivors who succeeded in escaping from the island. In addition were (2) personnel from the hotel that became the centre for victims and next of kin.

For each unit within the professional groups, a leader was appointed to distribute the questionnaires to personnel involved in the rescue work. The completed questionnaires were dropped anonymously into a sealed box. Some questionnaires were distributed by mail when this was more convenient. The municipality of Hole provided names and addresses for the unaffiliated volunteers, and the questionnaire was sent to them by mail with return envelopes. At the hotel, a leader distributed the questionnaires. For all groups, a reminder was sent after approximately 1 month. Returning the questionnaire was taken as implied informed consent. An information letter accompanied each questionnaire.

**Fig. 1:**
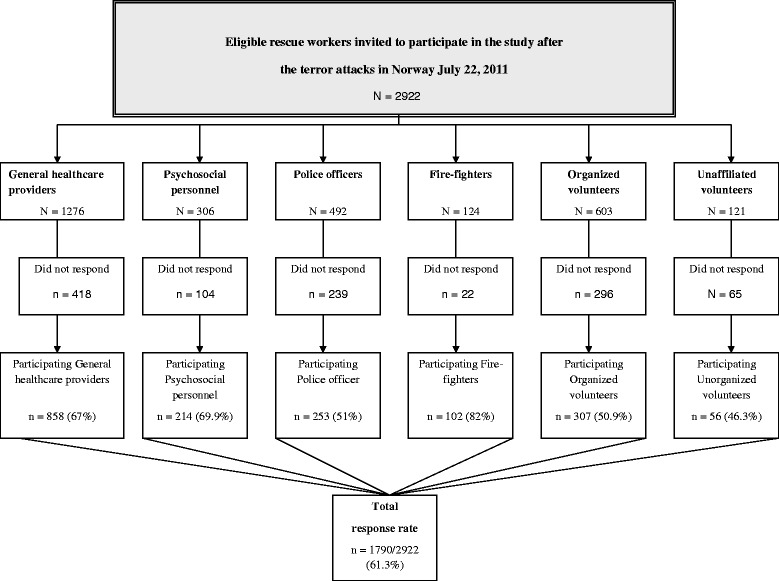
Eligible rescue workers invited to participate in the study after the terror attacks in Norway July 22, 2011 (*N* = 2922)

The self-assessment questionnaire was distributed between March and June 2012, approximately 8 to 11 months after the terror attack (mean 10 months). To obtain an overview of the response rate, each leader was asked to keep records of the number of distributed and returned questionnaires. Altogether, 2922 questionnaires were distributed (Fig. 1). The overall response rate was 61.3 %.

### Assessments

Most of the items in the questionnaire were developed by The Norwegian Centre for Violence and Stress Studies and used in a study of Norwegian personnel mobilized during the 2004 Indian Ocean tsunami [11].

#### Sick-leave

Sick-leave due to the terror-related work was assessed with response options: (a) no; (b) yes, <1 week; (c) yes, 1–2 weeks; and (d) yes >2 weeks. Items b–d were collapsed and coded as yes for the multivariable analyses.

#### Psychological help seeking

Psychological help seeking was assessed with one item: Have you made contact with a psychologist/psychiatrist or similar? Response options were no/yes.

#### Support

Support from the employer or organization in charge was assessed with the item: Did your employer/organization in charge provide support during the rescue work? There were five response options: (a) meetings such as defusing, debriefing or others; (b) meeting that marked transitions in work; (c) a psychologist/psychiatrist, priest or others; (d) gatherings; and (e) others. Participants could select more than one item. These items were merged and then dichotomized as no support offered or support offered.

#### Threat

Four items were used to assess possible perceived threats: Did you experience the following: (1) fear of explosion/shooting; (2) fear of being injured; and (3) other risks/uncertainties. Response options for each of these items were as follows: (a) 0 = no, not experienced; (b) 1 = yes, but not stressful; (c) 2 = yes, moderately stressful; and (d) 3 = yes, very stressful. The three items were summed and titled peritraumatic threat. The Cronbach’s alpha was 0.87.

#### Witnessing

Witnessing was measured using seven items. Witnessing disaster victims were as follows: those (1) searching for next of kin, (2) in despair at the campsite, (3) with major physical injuries, (4) seeing dead bodies, (5) having physical contact with dead bodies, (6) seeing body parts and (7) experiencing strong smells or other sensory perceptions. All items were dichotomized (no/yes). Their summed score reflected the number of positively scored items.

#### Psychological responses

Two questions assessed perceived psychological reactions: (1) did you feel overwhelmed and (2) did you feel that you had no control? These items were scored on a Likert scale 1–5 where 1 = not at all and 5 = to a very high degree.

#### The PTSD Checklist

The PTSD Checklist (PCL) is a widely used, self-administered measure of posttraumatic stress symptoms (PTSS) [16]. Seventeen items are scored on a Likert scale 1–5 from 1 = not at all to 5 = extremely. A score of ≥50 indicates possible PTSD, and a score of 35–49 indicates threshold PTSD. The validated Norwegian version was used [17].

#### Stress reactions

After the rescue work was ended: Did you experience unwanted and/or unexpected stress reactions? There were three response options: (a) yes, (b) no and (c) uncertain. Alternatives a and c were merged into yes = 1 and no = 0.

The other items and response options are shown in the tables.

### Statistics

The data are presented as means with 95 % confidence intervals or percentages. Variables were dichotomized where appropriate. Chi-square was used to compare descriptive data. To compare means independent sample tests were used on nonparametric data and ANOVA on normally distributed data. Logistic regression analysis (Forward Wald) was used to identify associations with sick-leave. Overall, there were few missing data (1–2 %) making corrections unnecessary. SPSS, version 21.0 (SPSS, Chicago, II), was used. The level for statistical significance was set at *p* < 0.05.

### Ethics

Oslo University Hospital’s Privacy Protection Supervisor and Vestre Viken Hospital Trust approved the study. The data were stored on the hospital research server. Approval from the Regional Ethics Committee was not required as the study was anonymous.

## Results

More women were found among the general and psychosocial healthcare providers compared to the other groups. Most of the rescue workers were between 30 and 49 years. The majority of the general healthcare providers, police officers and fire-fighters began work on July 22. They were all professional. Virtually all unaffiliated volunteers were “new recruits” on July 22, most of whom worked at the terror site landside of Utøya Island (Table 1).

**Table 1:** Background characteristics

*n* (%)	General healthcare providers	Psychosocial personnel	Police officers	Fire-fighters	Organized volunteers	Unaffiliated volunteers	*p* value
	*n* = 858	*n* = 214	*n* = 253	*n* = 102	*n* = 307	*n* = 55	
Gender							<.001**
Male	283 (33.1)	73 (34.1)	170 (68.0)	100 (99.0)	238 (77.5)	34 (63.0)	
Female	571 (66.9)	141 (65.9)	80 (32.0)	1 (1.0)	69 (22.5)	20 (37.0)	
Age							<.001**
<30 years	158 (18.5)	18 (8.5)	29 (11.6)	11 (10.8)	67 (21.8)	3 (5.6)	
30–49 years	534 (62.5)	90 (42.3)	172 (68.8)	68 (66.7)	193 (62.9)	34 (63.0)	
>50 years	162 (19.0)	105 (49.3)	49 (19.6)	23 (22.5)	47 (15.3)	17 (31.5)	
Previous training by simulation, yes	574 (67.8)	127 (59.3)	177 (70.0)	74 (72.5)	233 (76.1)	10 (18.2)	<.001**
Work location on July 22							<.001**
Sites of terror	138 (16.3)	0 (0)	92 (36.8)	99 (97.1)	170 (55.4)	34 (64.2)	
Others (hospital or municipal emergency services/centre for victims and next of kin/office, investigation, patrolling, security)	710 (88.7)	102 (47.7)	158 (63.2)	3 (2.9)	137 (44.6)	19 (35.8)	
Start work							
July 22, 2011	699 (81.9)	111 (51.9)	201 (79.8)	75 (74.3)	151 (49.2)	52 (96.3)	<.001*
July 23, or later	155 (18.1)	103 (48.1)	51 (20.2)	26 (25.7)	156 (50.8)	2 (3.7)	<.001*
Length of work							<.001*
<1 day	364 (42.8)	38 (17.8)	21 (8.4)	45 (44.1)	79 (26.0)	35 (66.0)	
1–7 days	286 (33.6)	103 (48.4)	123 (49.0)	47 (46.1)	200 (65.8)	15 (28.3)	
>8 days	200 (23.5)	72 (33.8)	107 (42.6)	10 (9.8)	25 (8.2)	3 (5.7)	

*p < .05; **p < .001

### Self-reported sick-leave

Among all groups, sick-leave was reported by 9.7 %; of those, 8.6 % reported sick-leave for less than 1 week (Table 2). The highest percentage of sick-leave for more than 2 weeks was reported by unaffiliated volunteers (5.5 %) compared with 0.0–1.2 % among the professionals. The rate of sick-leave varied from 2.4 % (police officers) to 14.5 % (unaffiliated volunteers), *p* < .001. More women (13.3 %) compared to men (6.5 %) reported sick-leave. A subgroup analysis of sick-leave among healthcare providers (general 13.9 % and psychosocial 12.8 %) showed that counsellors (e.g. priests, imams) (27.3 %), psychologists (23.1 %) and nurses/nurse assistants (15.6 %) reported the highest rates of sick-leave (Table 3).

**Table 2:** Exposure, sick-leave and help seeking among personnel within different occupations

mean (95 % CI) or *n* (%)	General healthcare providers	Psychosocial personnel	Police officers	Fire-fighters	Organized volunteers	Unaffiliated volunteers	*p* value
	*n* = 858	*n* = 214	*n* = 253	*n* = 102	*n* = 307	*n* = 55	
Peritraumatic threat (scale 0–3)	.5 (.5–.6)	.2 (.2–.3)	.8 (.7–.9)	.9 (.7–1.1)	.4 (.4–.5)	1.3 (1.0–1.6)	<.001**
Witnessing, number of witnessing experiences (0–7)	2.0 (1.9–2.1)	1.1 (1.0–1.3)	1.9 (1.7–2.2)	3.9 (3.6–4.3)	1.2 (1.0–1.3)	3.4 (3.0–3.9)	<.001**
Feeling overwhelmed (scale 1–5)	2.4 (2.4–2.5)	2.6 (2.5–2.8)	2.6 (2.5–2.8)	2.5 (2.3–2.7)	2.2 (2.0–2.3)	3.2 (2.8–3.5)	<.001**
Perceiving a lack of control (scale 1–5)	2.0 (2.0–2.1)	2.2 (2.1–2.3)	2.5 (2.3–2.6)	2.1 (1.9–2.3)	1.9 (1.8–2.0)	2.6 (2.2–2.9)	<.001**
Health-related problem at present caused by the rescue work							<.001**
Certainly not	691 (82.2)	160 (75.8)	178 (70.9)	79 (79.0)	258 (84.3)	21 (38.2)	
Probably not	134 (15.9)	43 (20.4)	58 (23.1)	19 (19.0)	43 (14.1)	16 (29.1)	
Yes, possibly/very likely	16 (1.9)	8 (3.8)	15 (6.0)	2 (2.0)	5 (1.6)	18 (32.7)	
Unwanted/unexpected stress responses (yes/uncertain)	253 (30.2)	65 (30.5)	68 (27.1)	33 (33.0)	72 (23.5)	34 (61.8)	<.001**
Contacted psychologist/psychiatrist	39 (4.8)	34 (16.3)	13 (5.1)	9 (9.1)	8 (2.6)	23 (42.6)	<.001**
Sick-leave							
Total	115 (13.9)	27 (12.8)	6 (2.4)	5 (5.1)	10 (3.3)	8 (14.5)	<.001**
0–7 days	110 (13.3)	25 (11.8)	2 (.8)	5 (5.1)	7 (2.3)	2 (3.6)	
1–2 weeks	3 (.4)	1 (.5)	1 (.4)	0 (0)	1 (.3)	3 (5.5)	
>2 weeks	2 (.2)	1 (.5)	3 (1.2)	0 (0)	2 (.7)	3 (5.5)	
Support provided from employer/crisis team (defusing/debriefing/psychologist/gatherings)	656 (81.2)	155 (74.2)	194 (80.5)	92 (94.8)	194 (64.7)	22 (50.0)	<.001*

**p < .001

**Table 3:** Sick-leave, stress responses and help seeking among healthcare providers and psychosocial personnel

*n* (%)	Physicians	Nurses and nurse assistants	Ambulance and emergency dispatchers	Other hospital personnel	Counsellors (priest, imam)	Social workers	Psychologists	*p* value
	*n* = 124	*n* = 502	*n* = 117	*n* = 139	*n* = 34	*n* = 24	*n* = 28	
Sick-leave	8 (6.7)	76 (15.6)	9 (7.7)	18 (13.7)	9 (27.3)	1 (4.2)	6 (23.1)	.004*
Unwanted/unexpected stress responses	14 (11.4)	152 (31.1)	46 (40.0)	42 (30.7)	10 (29.4)	10 (41.7)	7 (25.0)	<.001**
Posttraumatic stress symptoms (PCL) mean	18.5	19.9	21.3	21.1	20.1	20.0	19.5	<.001**
	(18.0–19.0)	(19.6–20.3)	(20.1–22.4)	(20.2–22.0)	(18.8–21.5)	(17.8–22.3)	(17.8–21.3)	
Contacted psychologist/psychiatrist	6 (4.8)	21 (4.2)	6 (5.4)	10 (7.4)	10 (29.4)	2 (8.3)	3 (10.7)	<.001**

*p < .05; **p < .001

### Psychological help seeking

Reported contact with a psychologist or psychiatrist after the rescue work differed significantly between groups. The highest was reported by unaffiliated volunteers (42.6 %) and psychological personnel (16.3 %) and the lowest by organized volunteers (2.6 %), *p* < .001. The organized (64.7 %) and unorganized (50 %) volunteers reported less provided support (debriefing, organized social gatherings, etc.) compared to professional rescue workers (74.2–94.8 %), *p* < .001 (Table 2).

### Peritraumatic responses

Fire-fighters (*M* = 3.9) and unaffiliated volunteers (*M* = 3.4) reported more witness experiences (people in despair and injured/dead) compared to the other groups (*M* = 1.2–2.0), *p* < .001. More peritraumatic threat was perceived among police officers (*M* = 0.8), fire-fighters (*M* = 0.9) and unaffiliated volunteers (*M* = 1.3) relative to the other groups (*M* = 0.2–0.5), *p* < .001. Perceived lack of control was reported significantly more often by police officers and unaffiliated volunteers. The latter group also reported a significantly higher mean score on feeling overwhelmed (Table 2).

### Stress reactions

About one third of respondents reported unwanted and/or unexpected stress reactions after the rescue work, with the highest percentage among unaffiliated volunteers (61.8 %), *p* < .001. After 10 months, 20 % of the unaffiliated volunteers still reported unwanted stress. This was higher compared to the professional rescuers.

The level of PCL was very low (*M* = 18–21) among all professional groups. In comparison, the unaffiliated volunteers reported a mean score of 33, *p* < .001.

### Associations with self-reported sick-leave

Nine univariate variables were significantly associated with sick-leave (Table 4). In the multivariable analysis, starting work on July 22 (OR 1.6) and unwanted/unexpected stress responses (OR 1.6) were independently associated with sick-leave. Psychosocial personnel (OR 6.3), general healthcare providers (OR 6.1) and unaffiliated volunteers (OR 5.7) were associated with sick-leave.

**Table 4:** Associations with sick-leave. (outcome: sick-leave no/yes)

Variables	Univariable (unadjusted)			Multivariable (adjusted)		
	OR	95 % CI	*p* value	OR	95 % CI	*p* value
Gender (man/woman)	2.2	1.6–3.1	<.001**			ns
Age			ns			
<30 years	ref					
30–49 years						
>50 years						
Previous training on simulation (no/yes)	.7	.5–.9	.013*			ns
Peritraumatic threat (continuous)			ns			
Witnessing (0–7)			ns			
Overwhelmed (continuous)			ns			
Lack of control (continuous)			ns			
Start work (July 23 or later/July 22)	2.0	1.3–3.0	.001*	1.6	1.0–2.5	.003*
Length of work <1-day ref						
1–7 days	.6	.4–.9	.005*			
>8 days	.6	.4–.9	.016*			
Working site (site of terror/others)	1.6	1.1–2.4	.010*			ns
Groups of rescue workers						
Police officers ref	ref					
Psychosocial personnel	6.0	2.4–14.5	<.001**	6.3	2.5–15.9	<.001**
General healthcare providers	6.6	2.9–15.1	<.001**	6.1	2.7–14.2	<.001**
Fire-fighters			ns			
Organized volunteers			ns			
Unaffiliated volunteers	6.9	2.3–20.9	.001*	5.7	1.9–17.4	.002*
Unwanted/unexpected stress reactions (yes and uncertain)	1.8	1.3–2.4	.001*	1.6	1.2–2.3	.004*

*p < .005; **p < .001; Logistic regression analysis (Forward Wald)

In the subgroup of healthcare personnel (hospital, emergency and psychosocial), the multivariable analysis with a physician as the reference group showed that being a nurse/nurse assistant (OR 2.6), psychologist (OR 6.0) and counsellor (e.g. priests, imams) (OR 6.4), along with starting work on July 22, (OR 1.9) was significantly associated with sick-leave.

## Discussion

The terror attacks in Norway, 2011, were demanding for rescue workers who had to handle chaotic situations. Despite this, the level of sick-leave for more than 2 weeks was 1.1 % among professionals and 5.5 % among unaffiliated volunteers. Although 9.7 % of the professional rescue workers reported sick-leave, only 6.1 % sought psychological help. The level of posttraumatic stress symptoms at 10 months was low, indicating that they needed only transient help. The unaffiliated volunteers reported more sick-leave than professional rescue workers and sought professional help more often. Psychosocial personnel, general healthcare providers and unaffiliated volunteers reported the most sick-leave. Other associations with sick-leave were starting work on July 22 and unwanted/unexpected stress reactions.

### Self-reported sick-leave

More general healthcare providers, psychosocial personnel and unaffiliated volunteers reported being on sick-leave. In 2012, the overall rate of sick-leave in Norway was 6.5 % (men 5.0 % and women 8.4 %) and 9.2 % among general healthcare providers and psychosocial personnel (men 6.1 % and women 9.9 %) [18]. In comparison, sick-leave among police officers was 2.9 % among men and 6.2 % among women. This supports that the rate of self-reported sick-leave due to the terror acts was low. Even though we have a cross-sectional design, the findings are comparable to those by Vázquez et al. [19]. They showed that initial symptoms were usually high in the first weeks after the attacks and returned to previous levels for most workers within weeks. However, Dirkzwager et al. found a slower decrease of sick-leave (6 months) after an explosion in the Netherlands in 2000 [1]. The Dutch study included only fire-fighters.

During and after the terror attacks in Norway, the surgical capacity was never exceeded in the emergency department, operating rooms or intensive care units, which may have been protective [13, 20]. Even so, treating young patients who had been hurt by human destructiveness caused unusual challenges. The impact was of short duration; still, this may have contributed to their strain. Counsellors (e.g. priests, imams) and psychologists reported the highest reported sick-leave. One explanation may be less experience with large-scale disasters, and they may have faced more unfamiliar tasks.

Sick-leave was also associated with starting work on July 22. This seems reasonable, as they were facing an unpredictable and potentially dangerous situation on the day of the attacks. The first responding rescue workers at the WTC on 9/11 reported more PTSD even 9 years after the terror attacks [21]. Thoresen et al. showed that the personnel coped well after the Indian Ocean tsunami in 2004, although personnel working in proximity to the disaster sites developed higher levels of stress reactions [11]. In the present study, being unprepared, threat and witnessing injuries, death and despair may explain the association between sick-leave and unaffiliated volunteers. The unaffiliated volunteers reported also significantly more perceived lack of control and less provided support compared to the professional personnel.

### Psychological help seeking

The terror attacks lasted for more than 3 hours, but the risk of more attacks was imminent during the first hours. Though a minority of rescuers worked at the site of terror, the situation felt threatening for most personnel during the first day. An additional risk factor for the development of posttraumatic stress symptoms, those who worked with patients and relatives over time had to face very traumatic stories. Most of the professional rescue workers were offered support, which may have had a preventive effect. Furthermore, the responders were mostly senior, and thus experienced.

We do not know how common psychological help seeking is among professional groups, but it is most likely less than the 6.1 % who sought help in the present study. Psychosocial personnel may have had easier access to psychological help and may be more familiar with processing personal psychological issues. This may partly explain why they sought psychological help more often. Fire-fighters and police officers, however, also had a 5–10 % rate of such help seeking, and these groups may be more reluctant to seek help for personal problems [22]. Though there may be several reasons for the low level of posttraumatic stress symptoms at 10 months, psychological help seeking may be a contributing factor in addition to the fairly short trauma duration and provided support. As very few were on sick-leave for more than 1 week, most of the psychological processing had probably been conducted after they returned to work. It is likely that most of the psychological healing occurred among peers at the workplace. In addition, the professional rescue workers were highly motivated; many showed up for work even before the disaster alarm went off, and some cancelled vacations.

The fact that 42.6 % of the unaffiliated volunteers in the present study sought psychological help underlines their massive exposure. In addition, few were trained and prepared for handling such tasks. This is in accordance with the findings in a review of volunteers [23]. The unaffiliated volunteers were the first on the scene. At the landside across from Utøya Island, they provided life-saving assistance while facing the possibility of being shot or suffering other injuries. Even after the site was secured, these workers were confronted with circumstances outside their usual experience. This probably resulted in unwanted stress reactions, with higher PCL scores, more sick-leave and more psychological help seeking relative to the professional groups. In addition, they reported less organized support, which may also partly explain these differences. The level of stress responses among the unorganized volunteers in the present study is more similar to victims than to professionals [23].

### Strengths and limitations

The response rate was satisfactory, and the high number of responders (*N* = 1790) was a strength despite varying response rates between groups. Few missing data was also a strength. The use of a well-established questionnaire such as the PCL-S strengthens the study. Interviews might have given additional information but would have required considerably greater investment. However, it is unlikely that an interview would have changed our main finding about sick-leave rates or that a considerable number of workers needed psychological help. Finally, it was a strength that different groups of professional rescue workers involved in the same terror acts were compared and also the inclusion of unaffiliated volunteers.

It was a study weakness that we had neither a control group nor sick-leave data for the groups, pre-attack. A prospective design would have given us the possibility to study changes over time.

### Clinical implications

Most of the professional rescue workers managed quite well. They quickly returned to work, and those in need seem to have sought help. The low levels of sick-leave and stress responses indicate that the system worked in terms of providing personnel support. In the future, the existing support system could be reinforced. More focus, however, should be given to the unaffiliated volunteers.

## Conclusions

A substantial number of workers sought psychological help after the terror acts in Norway, 2011, but their rate of sick-leave was low and of short duration. General healthcare providers and psychosocial personnel had the highest rates of sick-leave among the professionals, especially counsellors and psychologists. Stress reactions and starting work on July 22 were associated with more sick-leave. The unaffiliated volunteers reported significantly more sick-leave and psychological help seeking than the professionals. This group may benefit from more support.
